# Implants in Urogynecology

**DOI:** 10.1155/2015/354342

**Published:** 2015-04-29

**Authors:** Thomas Otto, Bernd Klosterhalfen, Uwe Klinge, Mihaly Boros, Dirk Ysebaert, Koudy Williams

**Affiliations:** ^1^Department of Urology, Lukas Hospital, 41464 Neuss, Germany; ^2^German Centre for Assessment and Evaluation of Innovative Techniques in Medicine (DZITM), 41464 Neuss, Germany; ^3^German Centre for Implant-Pathology, 52351 Düren, Germany; ^4^Department of Surgery, University of Aachen, 52074 Aachen, Germany; ^5^Department of Experimental Surgery, University of Szeged, Szeged 6720, Hungary; ^6^Department of Surgery, University of Antwerp, 2000 Antwerp, Belgium; ^7^Institute for Regenerative Medicine, Winston-Salem, NC 27101, USA

Implants aim to substitute or to support in case of own tissue deficiency and therefore played an increasing role for pelvic floor reconstruction in last decades. Several scientific theories (i.e., integral theory) and promising results from tertiary centers promoted the rising application of alloplastic materials [1]. With industry being deeply engaged in this field from the very beginning a huge variety of products was launched over the years and was approved due to simple administrative procedures (US: 510K; Germany/EU: medical product law and device regulation). Aggressive marketing helped to spread these products around the world, since there are millions of female patients in our aging society suffering from incontinence and pelvic floor prolapse. In a Public Health Notification (PHN), from 2008, the Food and Drug Administration (FDA) reported more than 1000 unexpected and severe adverse events, mostly associated with transvaginal placement of surgical mesh to treat pelvic organ prolapse (POP) and stress urinary incontinence (SUI). In 2011 and 2012, second and third FDA amendments were questioning the role of mesh application for POP and SUI repair and proposing a change to Class III status that would allow the request of premarket approval and postmarket surveillance studies [2]. A minority of patients (less than 5% according to Manufacturer and User Facility Device Experience MAUDE database) suffered from the complications but due to partly severe course and rising public interest the trend for mesh application stopped. Meanwhile, manufacturers in USA are confronted with >100 000 lawsuits. The allegations of the manufacturers are still severe. The companies are accused of misleading. The plaintiffs claim that it is “the legal duty of the manufacturers to ensure the efficacy and safety of transvaginal meshes,” but instead they provide patients with “false and misleading information about the efficacy and safety of products.” Several mesh products were withdrawn from the market. The majority of manufacturers are expected to compromise quickly with the plaintiffs, threatened by the numerous lawsuits and the bad course of the so-called “Bellwether Trials” for the industry.

Due to reported complications and consecutive law issues especially in USA there is a high uncertainty among clinicians and patients about the application of urogynecologic implants. New regulation strategies are urgent and in debate now.

There is a lack of appropriate preclinical tests and research on the risks of surgical meshes for use in female pelvic floor. However, what do we know by now? Tension-free vaginal tape (TVT) developed as a gold standard for the treatment of female SUI with good long-term functional results of 87% after 17 yrs of follow-up [3]; similar results were found for midterm follow-up of TOT (transobturator tape). Various single incision slings for female SUI and male slings are used for over 10 years but there is still a lack of good scientific data. In particular, there is a lack of knowledge to identify patients at high risk for mesh related complications, in which the risk benefit ratio is not balanced. Indication for pelvic floor devices should be focused on patients with low risk for mesh complications and high risk for failure of mesh-free procedures.

Artificial urinary sphincter is a gold standard therapy for severe male SUI but complete numbers of explanations (about 30%) and other complications are still missing. The current Cochrane Review included 19 randomised trials of anterior vaginal wall prolapse and found no significant differences for subjective postoperative outcome and quality of life, de novo dyspareunia, SUI, and reoperations with or without mesh-assisted reconstruction. Better anatomical results but more reoperations due to mesh erosions (10%) were found in the mesh-group [4]. The erosions are associated with other complications, as infections, bleedings, or chronic pain due to nerve lesions. The most severe complications include organ perforations, massive bleeding, and sepsis. Long-time complications are scarring and shortening of the vagina and recurrent POP and/or SUI.

What is the future for the use of alloplastic material for pelvic floor reconstruction?

Surgeons should perform surgery for SUI or POP only if they are adequately trained in this subspecialist area and are aware of all potential therapeutic options and complications. The surgeon experienced in the technique has less complications than less experienced surgeons [5].

The proper education of the patient is an obligation prior to operation. The pros and cons should be outlined for each patient prior to final selection of a surgical technique. Patients should be evaluated for risk factors and in case of recurrence the reasons for unsuccessful initial treatment and the feasibility of repeated surgical treatment should be evaluated [6]. The indications for meshes should be restricted to high-grade or recurrent prolapse, additional risk factors (obesity, lung emphysema, prolapse of multiple compartments, enterocele, and cystocele with lateral fascia defect). Postoperative control of results is important. A PF- (pelvic floor-) sonography is a very useful tool to control the mesh position. In case of complications or recurrence strategies mesh removal can be evolved based on PF-sonography [7]. Specialised centers should be consulted in case of complications; mesh removal is often a surgical challenge and can be associated with severe injuries and complications.

Material features like biocompatibility are crucial factors for foreign body reaction and ingrowth of the material. New studies for materials showed less complications if a type 1 mesh was used (monofilament, macroporous, and light-weighted) [8]. Further basic research studies on material features are important [9].

Finally, a structured development process and long-term registers for the implants are needed in order to provide better patient counselling and to promote technological improvements of alloplastic materials.

The current scientific framework, based largely on uncontrolled case series, does not serve patients well and has no future. In an era of comparative effectiveness, much stronger evidence and possibly cost-utility studies will be needed to evaluate treatment benefits and harms of the surgical therapies with the application of alloplastic materials.

In 2009, the Lancet dedicated a series to the topic of “Surgical Innovation and Evaluation” and its current status. A 5-stage description of the surgical development process was proposed, the so-called IDEAL model (innovation, development, exploration, assessment, and long-term study), which allows assigning every surgical innovation to its particular corresponding step of development (Table 1) [10, 11].

After the specification of the recommendations concerning IDEAL to the field of urology, several scientific groups utilized the IDEAL model of surgical innovation in the development of a novel surgical technique in order to show how surgical research may be concluded when strictly driven following standardized recommendations [11, 12].

A last stage of IDEAL requires long-term safety studies and registers. Up to date only a small number of implanted materials are evaluated in clinical trials. To provide the quality assurance of the medical products it is urgent to create national and international registers.

Such registers are already established by surgeons for inguinal and abdominal hernia repair (i.e., EuraHs) [13]. This knowledge could be implemented for the purpose of urogynecology by establishing a specialised implant register. The register would open the possibility to gain high quality results on indications, complications, and individual decisions concerning surgical methods and choice of material. Central registers are therefore the future instrument to provide the surgeon in a cost-effective and timely manner with the information for a responsible and individualized preoperative selection of the product.

The surgical procedures and the implementation of new techniques should be evaluated in consideration of human rights network and bioethical aspects. The Hippocratic oath “first do not harm” is a challenge in urogynecology and should lead every therapeutic decision.

We hope that the readers of this journal will find in this special issue not only accurate data and updated reviews on the recent development and indications for mesh application for POP and SUI but also the answer to such important questions as immunological and inflammatory reactions on the implantation of alloplastic materials and their impact on the biocompatibility in the host, latest imaging and other technologies for clinical evaluation and advances in biocompatibility of implants, next generation implants, strategies for clinical evaluation, and long-term surveillance of alloplastic materials (IDEAL).

*Thomas Otto* *Bernd Klosterhalfen* *Uwe Klinge* *Mihaly Boros* *Dirk Ysebaert* *Koudy Williams*

## Figures and Tables

**Table 1:** IDEAL stages (Dahm et al., 2014) [11].

Phase	Type of urologist involved	Purpose (study design)
Idea (1)	Very few, innovators	Proof of concept (structured case reports)

Development (2a)	Few, innovators and early adopters	Procedure development (prospective development studies)

Exploration (2b)	Many, early adopters and early majority	Refinement, community learning and consensus, and learning curve evaluation (preliminary collaborative cohort studies building toward randomized trials)

Assessment (3)	Many, early majority	Formal comparison of benefits and short-term safety (randomized controlled trials)

Long-term follow-up (4)	All eligible	Surveillance, quality assurance, and long-term safety (registry)
